# An Efficient Framework to Detect Intracranial Hemorrhage Using Hybrid Deep Neural Networks

**DOI:** 10.3390/brainsci13030400

**Published:** 2023-02-25

**Authors:** Manikandan Rajagopal, Suvarna Buradagunta, Meshari Almeshari, Yasser Alzamil, Rajakumar Ramalingam, Vinayakumar Ravi

**Affiliations:** 1 Department of CST, Madanapalle Institute of Technology & Science, Madanapalle 517325, India; 2 Department of CSE, Vignan’s Foundation for Science, Technology, and Research Vadlamudi, Guntur 522213, India; 3 Department of Diagnostic Radiology, College of Applied Medical Sciences, University of Ha’il, Ha’il 55476, Saudi Arabia; 4 Center for Artificial Intelligence, Prince Mohammad Bin Fahd University, Khobar 34754, Saudi Arabia

**Keywords:** intracranial hemorrhage detection, deep neural networks, deep RNN, CNN

## Abstract

Intracranial hemorrhage (ICH) is a serious medical condition that necessitates a prompt and exhaustive medical diagnosis. This paper presents a multi-label ICH classification issue with six different types of hemorrhages, namely epidural (EPD), intraparenchymal (ITP), intraventricular (ITV), subarachnoid (SBC), subdural (SBD), and Some. A patient may experience numerous hemorrhages at the same time in some situations. A CT scan of a patient’s skull is used to detect and classify the type of ICH hemorrhage(s) present. First, our model determines whether there is a hemorrhage or not; if there is a hemorrhage, the model attempts to identify the type of hemorrhage(s). In this paper, we present a hybrid deep learning approach that combines convolutional neural network (CNN) and Long-Short Term Memory (LSTM) approaches (Conv-LSTM). In addition, to propose viable solutions for the problem, we used a Systematic Windowing technique with a Conv-LSTM. To ensure the efficacy of the proposed model, experiments are conducted on the RSNA dataset. The suggested model provides higher sensitivity (93.87%), specificity (96.45%), precision (95.21%), and accuracy (95.14%). In addition, the obtained F1 score results outperform existing deep neural network-based algorithms.

## 1. Introduction

Damage or bleeding in the intracranial vault is referred to as intracranial hemorrhage (ICH). ICH detection is time-consuming even for highly experienced personnel [1]. Examining ICH diagnosis, on the other hand, is critical and time-consuming in order to pinpoint the bleeding site. As a result, an efficient and precise diagnosis is critical for the subsequent treatment process. To address this issue, some researchers hope to create neural networks capable of identifying hemorrhages based on a patient’s cranial CT scan. Neural network-based models may help radiologists diagnose ICH in a timely and accurate manner [2,3].

According to current studies, several research projects are being conducted that use deep learning algorithms for effective image categorization and segmentation. Most well-known deep learning systems, such as Convolutional Neural Networks (CNN), have piqued the interest of academics due to their ease of use and efficiency in dealing with medical diagnosis support [4,5]. CT scans are real-time 3D structures made up of a collection of 2D slices. Though working with image voxels is possible, it requires a high level of computing complexity. The latter can be avoided by processing slices independently or by utilizing the lowest 3D context complexity. Many deep-learning algorithms have been introduced in the literature to solve ICH detection and categorization. The authors of [6,7] presented a convolution neural network (CNN) to handle ICH challenges including segmentation and classification. However, the model’s precision is not adequate.

**Figure 1:**
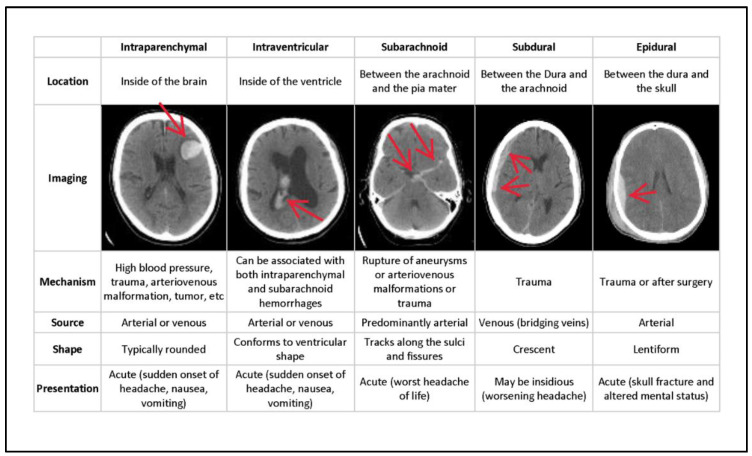
Types of hemorrhages and their respective location inside the skull [9].

In general, ICH is divided into six categories of hemorrhages. Epidural (EPD) pain occurs as a result of an injury to the dura mater and skull. Intraparenchymal (ITP) happens when blood pools in brain tissues; intraventricular (ITV) occurs when bleeding enters the brain’s ventricular system. SBC arises when blood gathers beneath the arachnoid and the pia mater. Subdural (SBD) bleeding occurs when blood flows between the dura mater and the arachnoid mater, and some (SOME) bleeding is defined as any hemorrhage [8]. Figure 1 depicts the many forms of hemorrhages, with the sixth being classified as SOME or ANY hemorrhage. The different types of hemorrhage occur in different areas of the skull.

Figure 1 depicts the type of bleeding, as well as the location and specifics of the hemorrhage. Even though the output from the neural network does not provide any location information, this is used to pinpoint the bleeding. Epidural (EPD), Intraparenchymal (ITP), Intraventricular (ITV), Subarachnoid (SBC), Subdural (SBD), and more designations are conceivable (SOME). If there is some hemorrhage, the final label will be correct. Multiple hemorrhages can occur at the same moment in some real-life settings [10].

The input for this task will be a single slice from a DICOM CT scan of a patient’s skull with dimensions of 512 × 512. The Dataset section goes into greater detail regarding the input data. For each label, the result will be a probability (between 0 and 1). Multiple hemorrhages can occur at the same time in real-world settings. As a result, the output labels are noy mutually exclusive. Each label is handled independently.

**Figure 2:**
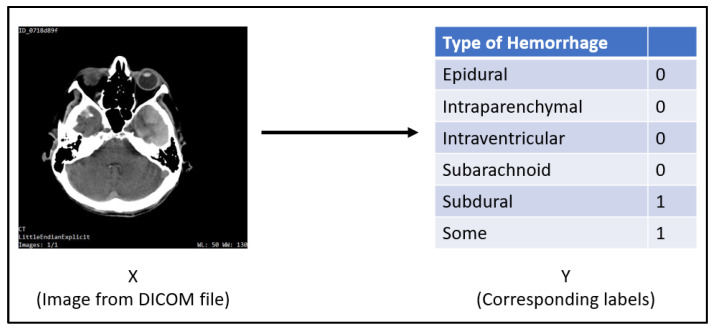
Illustration showing an example of Input/Output for the Neural Networks.

Figure 2 is a CT scan slice of a patient’s cranium showing s subdural hemorrhage as seen through the axial subdural window. It is mapped to its output, where subdural and some labels are 1 and the others are 0. A CT scan reveals some hemorrhage as well as a subdural hematoma.

This research presents a precise and well-organized method for analyzing single-slice 3D CT scan pictures using a standard neural network with systematic windowing. Only a few works that we are aware of use the systematic windowing technique, employing convolutional and recurrent layers for the ICH detection process. Very hybrid deep neural networks, however, are used directly on single-slice 3D CT scans. To successfully control ICH hemorrhage and produce an accurate outcome, a simple and efficient mixed Conv-LSTM approach is provided. We ensure that the proposed paradigm is simple in dealing with ICH concerns. Our primary contribution in this work is as follows.

- First, a hybrid deep neural network technique, convolution neural network and Long-short term memory (Conv-LSTM) for efficient and rapid identification of ICH in CT scans.
- Next, a systematic windowing approach is used to generate a temporal sequence. Conv-LSTM then extracts the spatiotemporal data and predicts it.
- Finally, the proposed model performance study shows that it is more accurate and has a lower false prediction rate than other existing learning approaches.

The next sections are structured as follows. The second section addresses recent research on ICH identification using deep learning algorithms. The dataset including RSNA Section 3 depicts intracranial hemorrhage and linear transformation, widowing and normalization, and the weighted sample method. The projected model, which resembles the process employed by radiologists in which the scan is systematically viewed under multiple window settings before an assessment is made, is covered in Section 4. Section 5 describes in depth the experimentation and findings after examining the recommended strategies. Finally, Section 6 concludes the study, followed by future directions.

## 2. Related Works

McCulloch and Pitts initially introduced artificial neurons in 1943 [11]. Later, this approach was enriched through growth in technology and system advances and has currently been utilized in various domains as a pioneering technology for image analysis.

The literature introduced numerous standard and profound learning approaches. Initially, many studies dealt with the ICH identification procedure using typical machine learning algorithms. The threshold-based technique for determining the ICH was introduced in [12]. The method identified ICH sub-types based on their position, volume, and contour. To optimize the threshold value, the author used retrospective instances of 33 CT scans and constructed their model using 210 CT scan images. The model achieved 59% specificity and 98% sensitivity for detecting ICH and moderate accuracy in classifying ICH sub-types. Li et al. [9,13] later introduced two ways to segment the SAH region and hemorrhage. One method uses elastic registration to deal with the SBC space atlas, while another employs the Bayesian decision strategy to extract distance transform features.

SAH space segmentation, entropy, mean grey value, energy, and variance, on the other hand, were defined and used to train the Support Vector Machine (SVM) classifier to detect SAH hemorrhage. The author used 60 CT images to train the model and 69 CT scans to test it. They discovered that the introduced model has a testing sensitivity of 100%, an accuracy of 91%, and a specificity of 92%.

Later, deep learning approaches drew the attention of various academics due to their ease of use and effectiveness in dealing with complicated problems. Arbabshirani et al. [14] used five layers and two fully linked CNN architecture models to detect the ICH over 46,583 records, which is one of the most practical strategies. These data are derived from official clinical radiology reports. The model’s performance achieves 96% accuracy and decreases the time required to diagnose new patient records. Prevedello et al. [15] presented two strategies based on the CNNs methodology. The early procedures focused on preventing ICH and hydrocephalus during a CT scan, while other methods were used to detect ICH sub-class kinds. For training and testing purposes, the author used a dataset of 319 records. For ICH detection, they specified 91% accuracy, 85% specificity, and 90% sensitivity. Grewal et al. [16] introduced CNN with an RNN model, RADnet, that uses standard DenseNet architecture for 3D slice level prediction. At the CT level, the author reported an 81.82% accuracy in ICH prediction. However, the model’s specificity and sensitivity are poor, and it can only handle a small dataset for prediction.

Jnawali et al. [17] created an ensemble learning model for ICH detection and segmentation using CNN architecture. The training model is trained using a dataset of 34,848 CT images, of which 8465 had ICH and 26,383 did not, though 5509 CT scans with and without ICH were used for testing purposes. The model’s performance showed 77% sensitivity, 80% precision, and 87% accuracy. However, after processing vast datasets, the model failed to improve ICH detection accuracy. [18] introduced a hybrid 3D CNN-RNN model that works directly on 3D CT images. They also used the Grad-CAM technique for image segmentation to process CNN attention maps. However, due to a restricted dataset for training purposes, the model faces significant challenges with a fresh CT scan.

Chan et al. [19] reported a knowledge-based classifier for detecting acute intracranial hemorrhage (AIH). A slice interpolation approach was developed to account for the spatial dependency among the closest slices. It reported 84% specificity, and 82% and 100% sensitivity at the slice and lesion levels, respectively. Shahangian et al. [20] created a method for image segmentation in CT scans called distance-regulated level set evolution (DRLSE). They first treated the segmentation to remove the irreverent forms that exist around the ventricles. They then carried out the ICH splitting utilizing DRLSE. On CT slices, the author reported 82.5% sensitivity and 90.5% specificity. Chang et al. [21] proposed convolutional neural networks based on region of interest (ROI). For training and testing, a dataset of 10159 and 862 records with and without ICH was used.

The majority of the studies published in the literature show inferior accuracy in the detection of some ICH subtypes, similar to the detection outcomes of low-grade radiology trainees [6]. However, recognizing SAH and EDH is a significant challenge for the various machine-learning methods [22,23]. To categorize the ICH sub-class type and improve classification accuracy, an efficient deep learning model is required.

## 3. Materials and Methods

### 3.1. Dataset: RSNA Intracranial Hemorrhage Detection

This dataset was provided by the RSNA (Radiological Society of North America) as part of a Kaggle competition called *RSNA Intracranial Hemorrhage Detection* [24]. The dataset is freely available for non-commercial and academic research purposes (see Competition Rules, point 7(A)).

The compressed data is 180 GB; the uncompressed data is 448 GB. It is divided into two sections: a labelled training set and an unlabeled test set. This study makes use of a dataset for training, validation and testing. Here, a single DICOM image file containing a single slice from a 3D CT scan is used. Exactly 269 files out of 752,803 had erroneous dimensions. They were narrowed down to 752,534 DICOM files. These are all the same size, 512 by 512 pixels. The statistics of the database are shown in Table 1.

Knowing how many files there are for each type of hemorrhage is also essential. We know the total number of files that show ICH hemorrhage, as shown in Table 2.

The important fact to remember here is that the total number of files (3145 + 36,115 + 26,204 + 35,675 + 47,060) equals 148,199. This is unusual because only 107,826 files reveal SOME bleeding (s). The 40,373-byte file has many hemorrhages. While counting, these files are counted many times, resulting in such an anomaly.

Now that we know that files display 1, 2, 3, 4, and 5 hemorrhages(s), we should look at how each is distributed in different files. There are now five “types” of files available (that show SOME hemorrhage). As a result, the class-wise distribution is as presented in Table 3.

The images were chosen carefully from this new data set for the neural networks we discuss in later sections.

### 3.2. Linear Transformation, Windowing and Normalization

DICOM files are not the same as standard files, such as JPEG or PNG images. DICOM files contain a lot of metadata, and the range of values for each pixel (voxel) is relatively broad when compared to normal image files.

The DICOM files were parsed using the Pydicom [25] package. To obtain the original Hounsfield values, a simple linear transformation is performed on the raw pixel array of the DICOM file.

(1) Hounsfield value=Raw pixel value×RescaleSlope+Rescale Intercept

The Rescale Slope and Intercept are in the DICOM file’s metadata.

After this linear transformation, an optional windowing might be performed. The window width and center values must be known to apply a window.

(2) $$\begin{array}{l} \mathrm{Min}=\mathrm{Window}\;\mathrm{Center}-\frac{\mathrm{Window}\;\mathrm{Width}}{2}\\ \mathrm{Max}=\mathrm{Window}\;\mathrm{Center}+\frac{\mathrm{Window}\;\mathrm{Width}}{2} \end{array}$$

(3) $$\mathrm{new}\;\mathrm{value}=\{\begin{array}{c} \mathrm{Min}\;\mathrm{if}\;\mathrm{value}<\mathrm{Min}\\ \mathrm{Max}\;\mathrm{if}\;\mathrm{value}>\mathrm{Max} \end{array}$$

After the optional windowing, pixel values are normalized to obtain a value between 0 and 1.

These steps are applied in training, validation and testing. This linear transformation and normalization should be performed even when making predictions with the model.

### 3.3. Weighted Samples

As described in Section 3.1, there is a significant class imbalance among various forms of hemorrhages. Class weights were employed to compensate for this mismatch and to allow the neural network to train more aggressively on infrequently observed hemorrhages.

### 3.4. Problem Formulation

Because the ground truth values are known, this is a supervised learning problem. Because the output classes are not mutually exclusive, this is a multi-label classification problem rather than a multi-class classification problem. Each output label is self-contained and functions as its own binary classification problem.

### 3.5. Selection of Appropriate Loss Function

Binary cross-entropy [26] is the chosen loss function taken element-wise over the entire output. The loss formula is given in Equation (4).

(4) loss=−(ylogp+(1−y)log(1−p))

where y is the ground truth value, and p is the predicted value.

This is taken element-wise over all the output labels and then averaged as per Equations (5) and (6).

(5) $$\begin{array}{c} {\mathrm{loss}}_{\mathrm{EPD}}=-\left(y_{\mathrm{EPD}}\times \log(p_{\mathrm{EPD}}\right)+\left(1-y_{\mathrm{SBD}}\right)\times \log\left(1-p_{\mathrm{SBD}})\right)\\ {\mathrm{loss}}_{\mathrm{ITP}}=-\left(y_{\mathrm{ITP}}\times \log(p_{\mathrm{ITP}}\right)+\left(1-y_{\mathrm{ITP}}\right)\times \log\left(1-p_{\mathrm{ITP}})\right)\\ {\mathrm{loss}}_{\mathrm{ITV}}=-\left(y_{\mathrm{ITV}}\times \log(p_{\mathrm{ITV}}\right)+\left(1-y_{\mathrm{ITV}}\right)\times \log\left(1-p_{\mathrm{ITV}})\right)\\ {\mathrm{loss}}_{\mathrm{SBC}}=-\left(y_{\mathrm{SBC}}\times \log(p_{\mathrm{SBC}}\right)+\left(1-y_{\mathrm{SBC}}\right)\times \log\left(1-p_{\mathrm{SBC}})\right)\\ {\mathrm{loss}}_{\mathrm{SBD}}=-\left(y_{\mathrm{SBD}}\times \log(p_{\mathrm{SBD}}\right)+\left(1-y_{\mathrm{SBD}}\right)\times \log\left(1-p_{\mathrm{SBD}})\right)\\ {\mathrm{loss}}_{\mathrm{SOME}}=-\left(y_{\mathrm{SOME}}\times \log\left(p_{\mathrm{SOME}}\right)+\left(1-y_{\mathrm{SOME}}\right)\times \log\left(1-p_{\mathrm{SOME}}\right)\right) \end{array}\;\;$$

(6) $$\mathrm{loss}=\frac{{\mathrm{loss}}_{\mathrm{EPD}}+{\mathrm{loss}}_{\mathrm{ITP}}+{\mathrm{loss}}_{\mathrm{ITV}}+{\mathrm{loss}}_{\mathrm{SBC}}+{\mathrm{loss}}_{\mathrm{SBD}}+{\mathrm{loss}}_{\mathrm{SOME}}}{6}$$

### 3.6. Model-Specific Details

Both models have a six-unit dense layer with sigmoid activation to ensure the output value for each label stays between 0 and 1.

(7) $$\mathrm{sigmoid}\;\left(x\right)=\frac{1}{\left(1+e^{-x}\right)}$$

Internal layers use ReLU as the activation function to make the training process faster.

(8) $$\mathrm{relu}\;\left(x\right)=\{\begin{array}{c} 0\;\mathrm{if}\;x\leq 0\\ x\;\mathrm{if}\;x>0 \end{array}$$

We employed the Adam optimizer [27] in our model. Glorot Uniform [28] is the kernel and bias initializer that eliminates the issue of exploding and endangered gradients due to the network being too deep. To prevent overfitting, L2 [29] regularization is utilized. Keras [30] was chosen as the framework, with Tensor flow 2.0 [31] serving as the backend.

## 4. Proposed Approach

By combining convolutional and recurrent layers, this study presented a hybrid deep convolutional LSTM model. Furthermore, the training procedure is divided into three primary parts. In the first stage, we use the systematic windowing method to simplify detection. The second stage involves training our CNN model to predict ICH types at the CT slice level. The CNN model identifies the important features and feeds them into the LSTM model [32]. We add the LSTM model for predicting ICH types at the CT scan picture in the third stage of the training procedure.

### 4.1. Systematic Windowing

The kernel *See like a Radiologist with Systematic Windowing* [33] by David Tang discusses viewing DICOM files under various window settings before making a prognosis. This is the same strategy that radiologists utilize when reviewing CT scans in real-world scenarios. DICOM files vary depending on the window settings; certain hemorrhages are considerably simpler to identify under certain window settings. Windowing is used specifically for this purpose, reported in Table 4. Based on this kernel and our understanding of the process, ten window settings were chosen.

**Figure 3:**
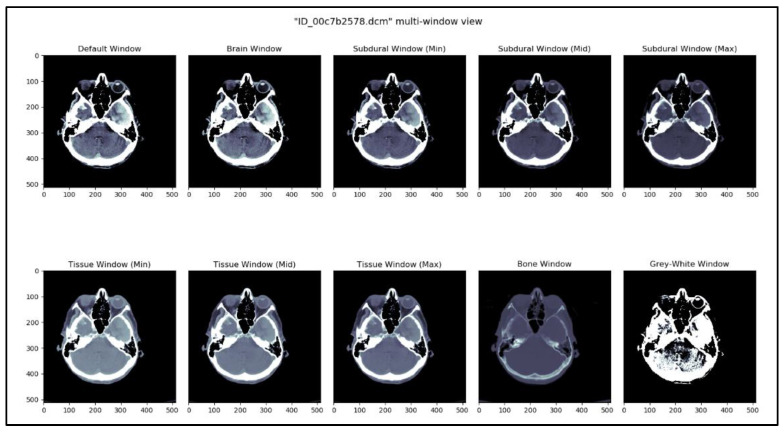
DICOM image viewed in 10 different window settings.

**Figure 4:**
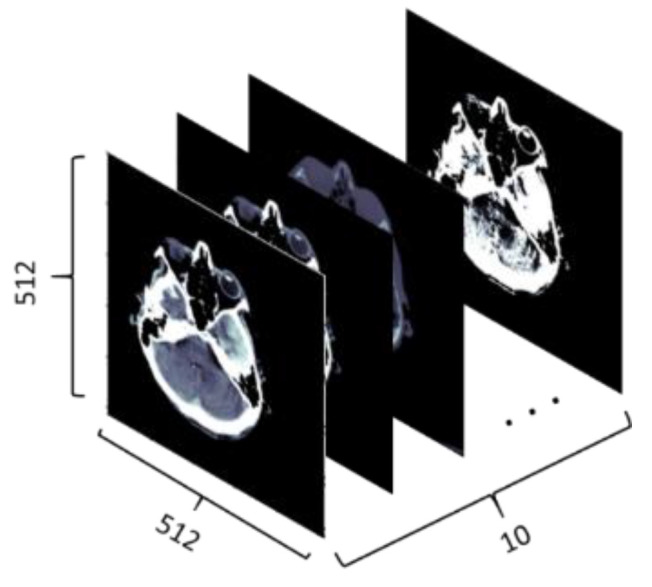
DICOM files under different window settings create a temporal sequence.

This methodology creates a temporal sequence. All window settings mentioned in Table 2 are applied, and the result is stacked. Figure 3 shows 10 different views of the DICOM image using different window settings. Figure 4 shows the creation of a temporal series taken as input by CNN. CNN extracts the spatiotemporal data and identifies the significant features. Later, the LSTM model utilizes the CNN model’s output and performs the accurate prediction level in the CT scan.

### 4.2. Convolutional Neural Network Model

**Figure 5:**
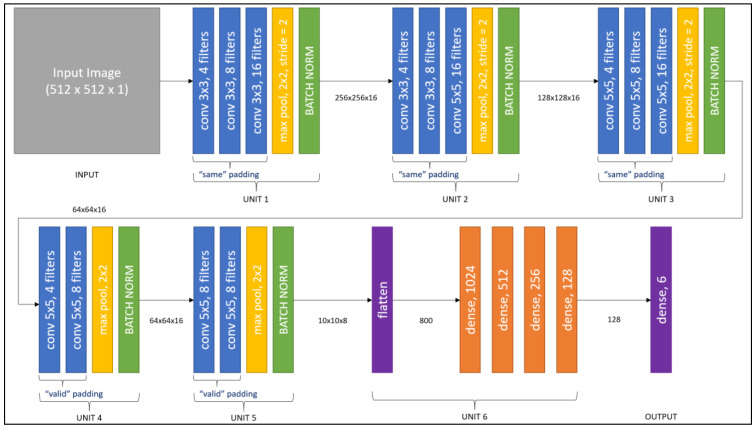
The working architecture of the CNN approach.

The CNN model is broken into six main units. The first four units are convolution, max-pooling, and batch normalization layers, with the final layer being fully-connected or dense layers. Figure 5 depicts each unit; unit 1 accepts a 512 × 512 × 1 NumPy matrix as input and includes three separate convolutional layers with 4, 8, and 16 filters of dimensions 3 × 3, 3 × 3, and 3 × 3, respectively. By employing the “same” padding, all of these convolution processes preserve the original extent. A max-pooling layer was used to decrease the image to half of its original size by utilizing a pool size of 2 × 2 and a stride of 2 × 2.

According to Figure 5, unit 2 receives the preceding unit’s output of dimension 256 × 256 × 16 as input and has three separate convolutional layers with 4, 8, and 16 filters of dimensions 3 × 3, 3 × 3, and 5 × 5, respectively. By employing the “same” padding, all of these convolution processes preserve the original extent. A max pooling layer was used once more to decrease the image to half of its original size by employing a 2 × 2 pool size with a stride of 2 × 2. Unit 3 receives the preceding unit’s output of size 128 × 128 × 16 as input and has three distinct convolutional layers with 4, 8, and 16 filters of dimensions 5 × 5, 5 × 5, and 5 × 5, respectively. By employing the “same” padding, all of these convolution processes preserve the original extent. A max pooling layer was utilized once more to decrease the image to half of its original size by employing a 2 × 2 pool size with a stride of 2 × 2.

Unit 4 in Figure 5 takes the preceding unit’s output of dimension 64 × 64 × 16 as input and has two convolutional layers with 4 and 8 filters of dimensions 5 × 5 and 5 × 5, respectively. Using a 2 × 2 filter, a max-pooling layer was utilized to compress the image to half of its original size. Unit 5 accepts as input the previous unit’s output of dimensions 28 × 28 × 8. It contains two convolutional layers with 8 and 8 filters in 5 × 5 and 5 × 5 dimensions, respectively. A max-pooling layer helps to decrease the image to half of its original size when applying a 2 × 2 filter. By using unit 6, the output from the preceding unit is converted to an input of 800. Following that, there are four dense layers of 1024, 512, 256, and 128 units, respectively. The final/output layer has a thickness of 6 units. In the final layer, the activation function is sigmoid. The final shape is (6).

### 4.3. Deep Conv-LSTM Model

LSTMs are recurrent neural network variants that can handle data point sequences. LSTM networks have been used by several academics to handle complicated problems such as language modelling, machine translation, and others. The merging of CNN and LSTM is introduced in this study by mining key features from CT images using CNN and then providing input features to the LSTM. The LSTM then takes the feature sequences as input and predicts using the entire CT scan. Long short-term spatiotemporal relations can be mapped using LSTMs. As a result, it simulates a radiologist going through numerous windows before making an assessment.

**Figure 6:**
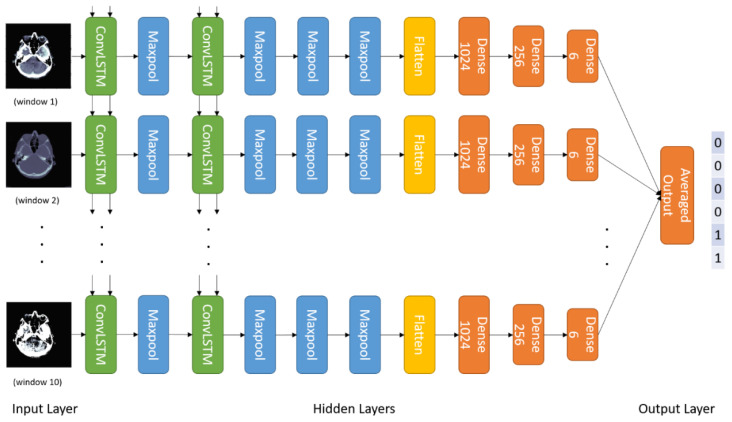
The architecture of the Conv-LSTM Model for ICH Detection.

Figure 6 depicts the entire architecture of our proposed model. The CNN model retrieves features from the CT image using the 10-window slice input. The features are then incorporated into the LSTM model, which appropriately classifies the ICH hemorrhage kinds.

### 4.4. Training and Testing

In this paper, we chose a training dataset of 72,516 CT scans for training purposes. The training dataset contains a variety of hemorrhages. Furthermore, the initial sample weights were used to account for the changing ratios in which the photos appeared. The model was trained on the chosen dataset for 500 epochs. Based on the results of the numerous runs, we decided on 500 epochs. because the model stops learning after 500 epochs and produces the same result. The performance of the proposed model is measured every 100 epochs to confirm that the learning process is improving. The suggested network is clearly correcting itself to recognize negatives more correctly than previously. In contrast, we chose 7515 CT scans for validation and 7512 CT scans for testing. The CT scan pictures chosen for training, validation and testing are used in other models to compare the performance of the proposed model.

## 5. Experimentation and Results Analysis

This section covers the performance indicators that will be used to validate the proposed approach. Furthermore, the experimental results and analysis of results with other existing models are discussed here.

### 5.1. Performance Metrics

To validate our proposed approach, we used major performance metrics. The classification performance was assessed using the metrics listed below.

(a) Sensitivity (also known as True Positive rate (TPR)):
  $$\mathrm{TPR}=\frac{\mathrm{TP}}{\mathrm{TP}+\mathrm{FN}}\times 100\%$$
(b) Specificity (also known as True Negative rate (TNR)):
  $$\mathrm{TNR}=\frac{\mathrm{TN}}{\mathrm{TN}+\mathrm{FP}}\times 100\%$$
(c) Accuracy:
  $$\mathrm{Acc}=\frac{\mathrm{TP}+\mathrm{TN}}{\mathrm{TP}+\mathrm{FP}+\mathrm{TN}+\mathrm{FN}}\times 100\%$$
(d) F1 Score:

$$F1=\frac{2\times \mathrm{TP}}{2\times \mathrm{TP}+\mathrm{FP}+\mathrm{FN}}\times 100\%$$

where TP and TN specify the true positive and negative rates, and FP and FN specify the false positive and negative rates, correspondingly.

### 5.2. Experimental Results

The experiment was carried out using a PC equipped with an i5 processor running at 2.3 GHz, 16 GB RAM, and 500 GB SSD file storage, and the dataset was obtained from the Kaggle repository. Furthermore, we employed the Python 3.8 tool together with significant libraries, such as Tensorflow, Keras, NumPy, Matplotlib, and OpenCV-python. A collection of 87,543 files contained 512 × 512 pixels from patients’ CT scans [24]. We chose 72,516 CT scans for training and 7515 and 7512 CT scans for validation and testing from the given dataset. The outcomes are measured using the standard performance measures described in Section 5.1. The suggested model’s performance is compared to that of state-of-the-art techniques, including ResNexT [34], SVM, CNN [35], AlextNet + SVM [36], U-Net, and WA-ANN [37]. The Adam optimizer was used, employing a binary cross entropy loss and a learning proportion of 0.0001.

**Figure 7:**
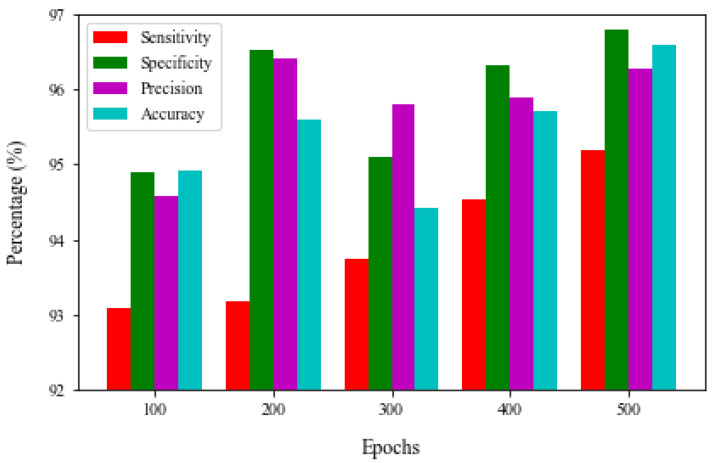
Proposed Conv-LSTM model outcome under varying epochs.

Table 5 and Figure 7 show the results of the Conv-LSTM model after 500 epochs. We discovered that the proposed model has a maximum TPR of 92.09%, TNR of 94.90%, precision of 94.57%, and accuracy of 94.91% after analyzing the classifier output across 100 generations. On the other hand, the results of 200 epochs show that the suggested model achieves 93.18% TPR, 96.52% TNR, 95.10% precision, and 95.60% accuracy. Simultaneously, the results under 300 epoch show a TPR of 92.75%, TNR of 95.10%, precision of 95.79%, and accuracy of 94.43%. Under 400 generations, the suggested model inevitably obtains a TPR of 94.54%, TNR of 95.31%, precision of 94.90%, and accuracy of 95.70%. The suggested Conv-LSTM technique yields an optimal TPR of 95.20%, TNR of 96.80%, precision of 96.27%, and accuracy of 95.70%.

**Figure 8:**
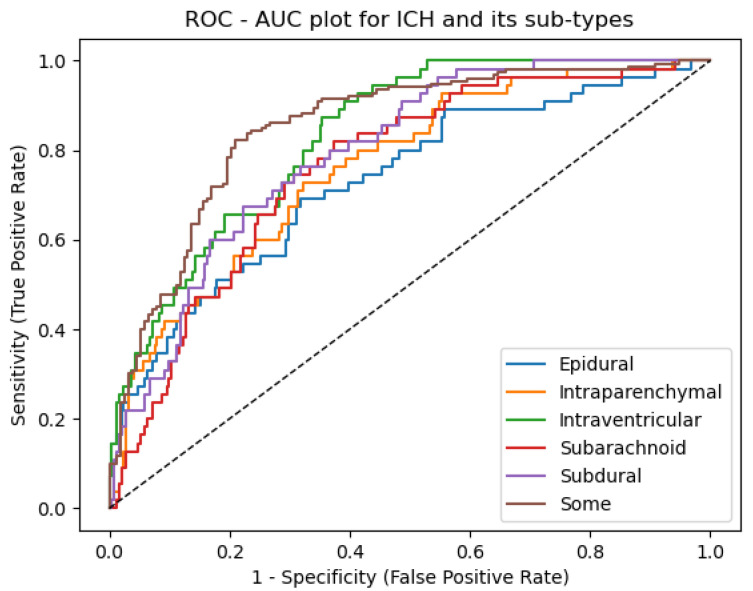
ROC-AUC plot for ICH and its sub-types.

Figure 8 depicts the outcome of the proposed model’s ROC (receiver operating characteristic) curve. It depicts the network’s performance at various thresholds. The AUC (area under the curve) score was determined separately for each of the labels listed in Table 6. The proposed model has a better ROC-AUC score for some of 94.74%, EPD of 93.11%, ITP of 95.13%, ITV of 93.06%, SBC of 95.11%, and SBD of 95.37% for classifying the ICH sub-class.

### 5.3. Comparative Result Analysis

**Figure 9:**
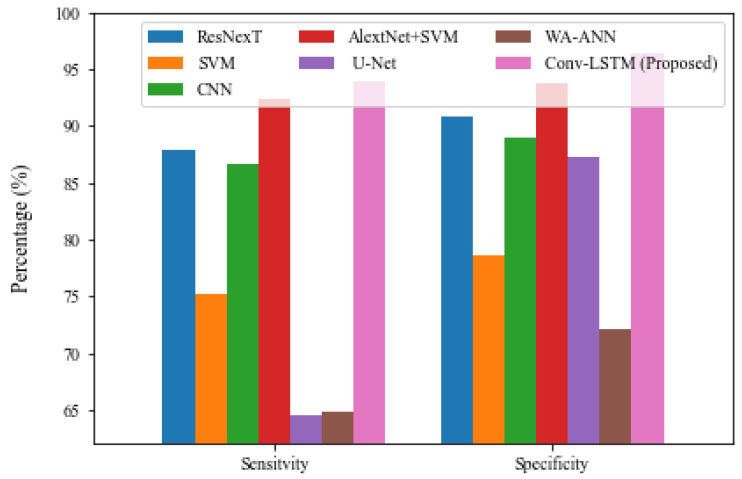
Comparative result analysis of Conv-LSTM with Sensitivity and Specificity.

Table 7 presents a comprehensive compared result analysis of the proposed model with other state-of-the-art existing methodologies. Figure 9 depicts the sensitivity and specificity of the proposed model and alternative models. The WA-ANN model resulted in low performance in terms of TPR and TNR, with 64.87% and 72.15%, respectively, whereas U-Net performed somewhat better than WA-ANN, with sensitivities of 64.54% and 87.21%, respectively. As a result, the SVM technique produced better results, with a TPR of 75.12% and a TNR of 78.64%. Furthermore, the CNN model attempted to deliver superior results, with a TPR of 86.61% and a TNR of 88.94%. At the same time, the ResNexT model produced a reasonable classification result with a sensitivity of 87.84% and a specificity of 90.78%. Though the AlexNet+SVM model produces superior results with specificity and sensitivity of 92.45% and 93.74%, respectively, the suggested model outperforms all other models with sensitivity of 93.87% and 96.45%. It is stated unequivocally that the suggested model achieves correct classifications and achieves higher values of TPR, TNR, FPR, and FNR on the tested examples.

**Figure 10:**
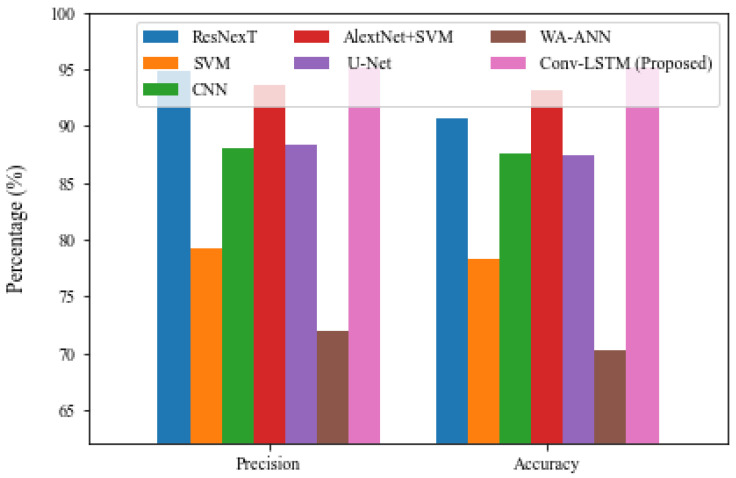
Comparative result analysis of Conv-LSTM with Precision and Accuracy.

Figure 10 depicts the precision and accuracy attained by the proposed model and the other models compared. The graphic illustrates that the existing WA-ANN and SVM models do not perform better on the test case photos. WA-ANN and SVM models achieved precision of 71.94% and 79.27%, respectively, while WA-ANN and SVM projected accuracy of 70.27% and 78.24%, respectively. This demonstrates that classifying these two models does not produce superior results. Furthermore, the U-Net and CNN models produced results with precision of 77.39%, 87.98%, and accuracy of 87.50% and 87.63%, respectively. Although these two models attempted to produce better results, they were unable to categorize the photos containing the bleeding.

Furthermore, the ResNexT and AlexNet+SVM models tried to provide a competitive outcome with a precision of 94.86%, 93.67% and accuracy of 90.71% and 93.18%; the proposed model has surpassed the other existing approaches with a precision of 95.21% and accuracy of 95.14%, respectively. From the comparative results, we observed that the proposed model achieved better outcomes; thereby, the proposed Conv-LSTM model is a suitable technique for ICH diagnosis. The superior result of Conv-LSTM is due to the significant feature extraction and selection using the CNN model and LSTM-based classification.

### 5.4. Discussion

Conv-LSTM produces balanced outcomes with primarily minor differences. Conv-LSTM architecture is a hybrid of CNN and LSTM models, with EPD and ITV classification performing most and least efficiently, respectively. Except for the EPD, the CNN has a minor edge over the LSTM. Keep in mind, however, that the EPD and ITV provide much poorer classification results than the other subtypes. The subtype-classification-assessment ranking represents the order produced by individual ResNets. Nonetheless, the proposed architecture improves classification in each category. According to Table 6, the increases in ROC-AUC score vary by subtype, ranging from approximately 0.1–0.5% in Some, ITP, SBC, and SBD to more than 1–2% in EPD and ITV, which have the most space for advancement. The regularity of performance across distinct ICHs leads us to infer that using an external learning classifier with hybrid deep features is beneficial in ICH detection.

The usage of multi-source features for classification can be linked to improved performance of the proposed Conv-LSTM architecture. The combination of features retrieved by networks addressing specific picture data representations yields a more comprehensive set of data. A slice-by-slice neighborhood context appears to be very important. If the entire CT scan is arranged correctly, bleeding should be seen in the following slices. As a result, such geographical information boosts the automated system’s cognitive capacities.

Slice-wise brain hemorrhage detection frameworks typically operate on the full CT slice or, in the case of our technique, conduct some primary ROI extraction to prepare the data for analysis. The deep learning tool handles the majority of the processing, with the operator having little influence on feature extraction. Some focused localization of specific areas of the image may boost detection and classification results. The differences in hemorrhage subtypes are related to their location and morphology. Considering the foregoing in more detailed specialized processes may aid categorization accuracy improvement. Mechanical head traumas frequently result in hemorrhages. In addition to the deep learning analysis, the external area of the skull might be analyzed.

The lowest metrics obtained in EPD classification highlight concerns about class imbalance in a single classification challenge. The number of epidural cases is significantly lower than those of other kinds. That is most likely the case in this example of bleeding, and the class weighing used during training is insufficient to remedy the problem. However, no such association exists across all subtypes. The ITP with the highest classification accuracy (detecting 29 out of 30 instances correctly) is not the best-represented hemorrhage; the second-best ITV (14 out of 15 right) is the second-worst-represented ICH subtype. Thus, the presentation of a specific hemorrhage in CT has a significant impact on detection skills. Similarities between various bleeding symptoms and characteristics are also possible (recall that the dataset of negative samples in each subtype classification also contains images with all other hemorrhages). Some complex examples exhibit a mix of numerous kinds at the same time. As a result, correct classification is surely a difficult assignment.

Table 8 summarizes the F1 score values from relevant state-of-the-art ICH detection approaches, as well as our proposed Conv-LSTM. Because it ignores true negatives, the F1 score is a credible metric in severely imbalanced datasets. The approaches used addressed at least half of our bleeding subtype set detection. Most crucially, they found hemorrhages in CT slices rather than the complete 3D series, as we did. The RSNA competition database was used by Burduja et al. [8] and Danilov et al. [38]. We used this to supply data and evaluation results to calculate the F1 score when it was not clearly stated.

## 6. Conclusions

This work contributes to the investigation of CNN and LSTM techniques for determining intracranial hemorrhage (ICH) within the skull. The research demonstrates that the network can predict the type of ICH found in a CT scan. This is seen from the network’s sensitivity, specificity, and accuracy over predictions on the testing data. It is equally effective in avoiding false positives and false negatives, which is a major issue in medical testing. The ROC-AUC ratings for each label show that the proposed study detects certain types of hemorrhages. This network uses a 2D slice from a 3D scan to predict in all of the slices. It allows us to evaluate the full brain scan, examine each section separately, and determine the ICH class kinds. Experiment findings show that the proposed model has a sensitivity of 93.87%, a specificity of 96.45%, a precision of 95.21%, and an accuracy of 95.14%. The proposed Conv-LSTM model outperforms the competing models. The work can be expanded further by introducing an optimization algorithm to select the optimal features and to train the model to determine the optimal weight classifier.

## Figures and Tables

**Table 1:** The total number of files with and without hemorrhage (s).

Files that show some hemorrhage(s)	107,826
Files that do not show any hemorrhage	644,708
Total files	752,534

**Table 2:** The number of files in ICH hemorrhage.

Hemorrhage	Total Number of File(s) with This Hemorrhage Present in Them
SOME	107,826
EPD	3145
ITP	36,115
ITV	26,204
SBC	35,675
SDB	47,060

**Table 3:** The number of files of each file type per hemorrhage.

Hemorrhage	Number of Files of Each Type for This Hemorrhage					Total
	1	2	3	4	5	
EPD	1694	923	368	137	23	3145
ITP	15,664	14,338	5112	978	23	36,115
ITV	9878	11,617	3730	956	23	26,204
SBC	16,423	12,911	5329	989	23	35,675
SBD	32,096	9857	4112	972	23	47,060

**Table 4:** Different window settings.

Window Name	Window Center (WL/WC)	Window Width (WW)
Default	from DICOM tags	from DICOM tags
Brain	40	80
Subdural (min)	50	130
Subdural (mid)	75	215
Subdural (max)	100	300
Tissue (min)	20	350
Tissue (mid)	40	375
Tissue (max)	60	400
Bone	600	2800
Grey-white differentiation	32	8

**Table 5:** The outcome of the proposed Conv-LSTM model.

Epochs	Sensitivity/TPR (%)	Specificity/TNR (%)	Precision (%)	Accuracy (%)
100	92.09	94.90	94.57	94.91
200	93.18	96.52	95.10	95.60
300	92.75	95.10	95.79	94.43
400	94.54	95.31	94.90	95.70
500	95.20	96.80	96.27	96.58

**Table 6:** ROC-AUC score for different labels.

Metric	Labels					
	Some	EPD	ITP	ITV	SBC	SBD
ROC-AUC Score (%)	94.74	93.11	95.13	93.06	95.11	95.37

**Table 7:** Comparative result analysis of proposed model with other state-of-art techniques.

Algorithms	Sensitivity/TPR (%)	Specificity/TNR (%)	Precision (%)	Accuracy (%)
ResNexT	87.84	90.78	94.86	90.71
SVM	75.12	78.64	79.27	78.24
CNN	86.61	88.94	87.98	87.63
AlextNet + SVM	92.45	93.74	93.67	93.18
U-Net	64.54	87.21	88.39	87.50
WA-ANN	64.87	72.15	71.94	70.27
Conv-LSTM (Proposed)	93.87	96.45	95.21	95.14

**Table 8:** Comparison of classification F1 score achieved by proposed technique and other compared techniques.

Algorithms	Some	EPD	ITP	ITV	SBC	SBD
ResNexT	88.74	84.51	89.69	85.12	90.71	91.62
SVM	74.88	72.94	77.17	73.84	79.14	79.67
CNN	88.91	83.74	87.16	83.51	88.37	87.38
AlextNet + SVM	92.17	90.45	92.67	91.47	93.08	92.87
U-Net	84.17	82.88	86.74	77.97	87.18	86.24
WA-ANN	72.15	72.14	72.74	71.86	72.64	71.45
Conv-LSTM (Proposed)	94.63	93.15	94.87	93.16	95.61	95.37

## Data Availability

Dataset (https://www.kaggle.com/c/rsna-intracranial-hemorrhage-detection/data, accessed on 14 September 2022) is taken from RSNA Intracranial Hemorrhage Detection [1] competition on Kaggle. Used under point 7(A) of Competition Rules [2] (B. General Competition Rules) stated at https://www.kaggle.com/c/rsna-intracranial-hemorrhage-detection/rulesfor the purpose of academic research.
